# Morphology regulation in vascular endothelial cells

**DOI:** 10.1186/s41232-018-0083-8

**Published:** 2018-09-10

**Authors:** Kiyomi Tsuji-Tamura, Minetaro Ogawa

**Affiliations:** 1 Department of Cell Differentiation, Institute of Molecular Embryology and Genetics, Kumamoto University, Kumamoto, 860-0811 Japan; 2 Present Address: Oral Biochemistry and Molecular Biology, Department of Oral Health Science, Faculty of Dental Medicine and Graduate School of Dental Medicine, Hokkaido University, Sapporo, 060-8586 Japan

**Keywords:** Vasculature, Endothelial cells, Angiogenesis, Morphology, Elongation

## Abstract

Morphological change in endothelial cells is an initial and crucial step in the process of establishing a functional vascular network. Following or associated with differentiation and proliferation, endothelial cells elongate and assemble into linear cord-like vessels, subsequently forming a perfusable vascular tube. In vivo and in vitro studies have begun to outline the underlying genetic and signaling mechanisms behind endothelial cell morphology regulation. This review focuses on the transcription factors and signaling pathways regulating endothelial cell behavior, involved in morphology, during vascular development.

## Background

### Vascular system development

**Fig. 1:**
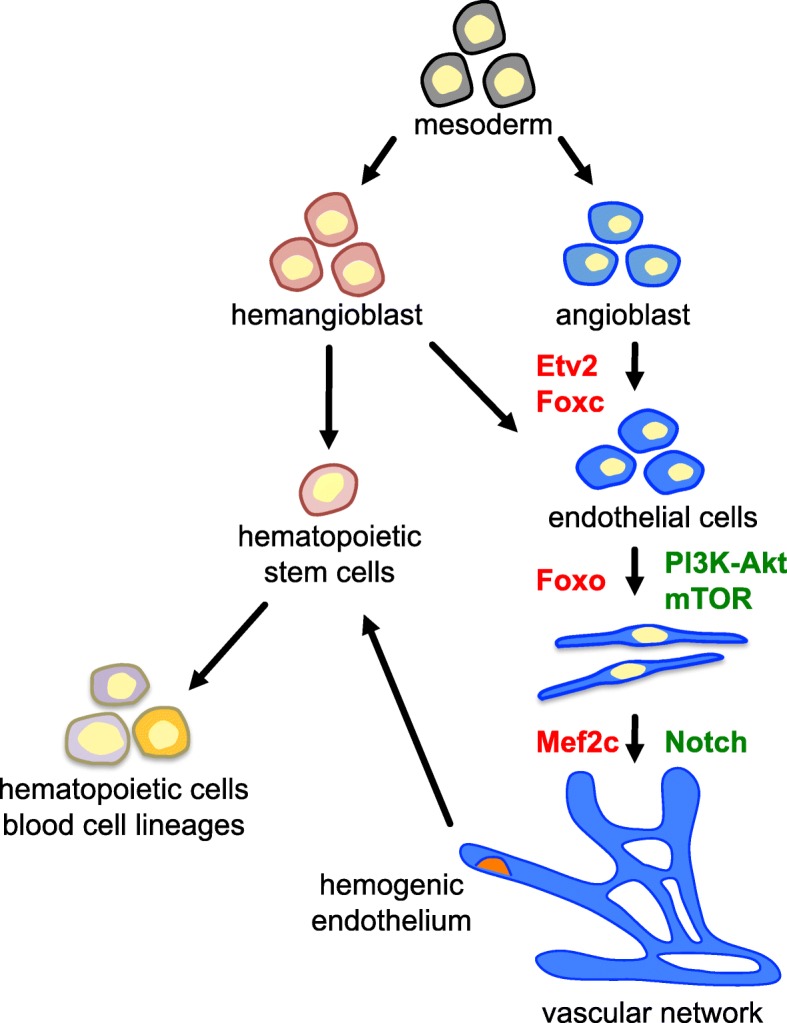
Schematic model of early vascular development. Endothelial cells are derived from mesodermal precursors: angioblasts and hemangioblasts. They form vascular networks by undergoing morphological changes. Possible transcription factors (red) and signaling molecules (green) controlling each process are shown. During early vascular development, hematopoietic lineages arise from hemangioblasts or hemogenic endothelium

During the earliest stages of embryonic development, vascular formation occurs in connection with blood cell formation (hematopoiesis) [1, 2]. There are various theories about the origin of endothelial cells, but the mesoderm has been reported to generate an endothelial cell progenitor (angioblast) and a common progenitor of hematopoietic cells and endothelial cells (hemangioblast) [3] (Fig. 1). De novo vascularization, or vasculogenesis, is accomplished by endothelial cells derived from these mesodermal progenitors. During this process, cells form a primitive vessel network that serves as the basis for the mature vascular system [4]. New blood vessels are then formed from pre-existing ones and spread into avascular areas. This process, in which the network of early primitive vessels is expanded, is defined as angiogenesis [5]. Subsequently, vasculature undergoes remodeling in an ordered manner. Initiation of endothelial cell specification into arteries and veins appears to occur before forming structural arteries and veins [6]. Vasculature maturation results when new blood vessels recruit and are linked to vascular smooth muscle cells and pericytes. In addition, a population of endothelial cells known as the hemogenic endothelium reportedly generates hematopoietic stem cells directly [3, 7–10].

Specification of angioblasts to either arterial or venous endothelial cells is established prior to forming blood vessel structures [11–13]. The receptor tyrosine kinase EphB4 and its transmembrane ligand ephrinB2 are demonstrated to be significant factors for arteriovenous definition [14]. The binding of vascular endothelial growth factor (VEGF) to its receptor VEGFR2, also known as KDR/Flk1, induces the expression of ephrinB2 through Notch signaling in arterial-fated precursor cells [15]. The specification of venous endothelial cells appears to set as the default in the absence of Notch signaling. Moreover, it has been reported that chicken ovalbumin upstream promoter-transcription factor II (COUP-TFII), which specifically expressed in venous endothelial cells, suppresses Notch signaling, leading in maintain vein identity [16]. After that, a subpopulation of venous endothelial cells acquires the expression of prospero homeobox 1 (Prox1) transcription factors, leading to specification of lymphatic endothelial cells [13, 17, 18]. COUP-TFII directly interacts with Prox1 and also controls lymphatic cell fate [19].

**Fig. 2:**
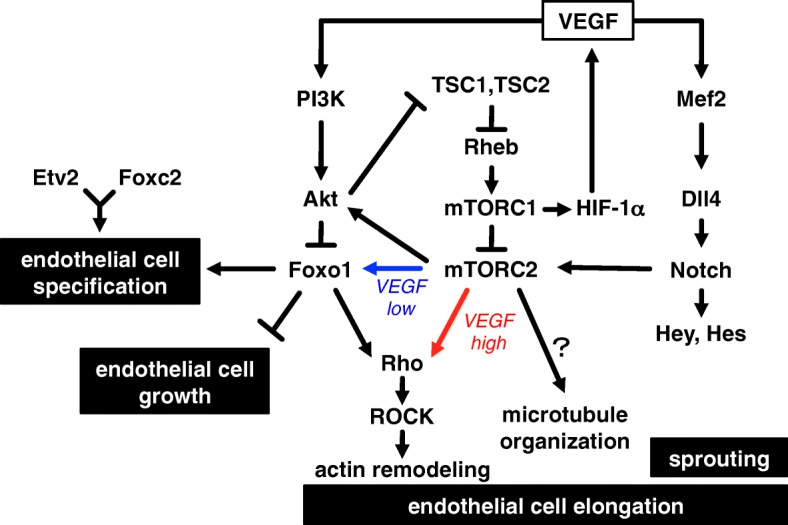
Schematic model of transcription factor and signaling molecule interactions in endothelial cell functions. VEGF regulates endothelial cell functions through interaction and association with PI3K-Akt, mTOR, and Notch signaling. Foxo1-dependent (blue) and Foxo1-independent (red) pathways for endothelial cell elongation are shown. Pathway depends on environmental levels of VEGF

The process of vascular development requires various and complicated endothelial cell angiogenic behaviors. As endothelial cells proliferate, migrate, and undergo morphological changes such as elongating and sprouting, they assemble into a solid linear mass called a vascular cord. Following this, tubulogenesis occurs through lumen formation at the center of the cord [20]. These processes are orchestrated at the genetic and signaling levels [21, 22]. In this review, we concentrate on transcriptional regulators and signaling pathways required for endothelial cell regulation, especially on morphology, during vascular formation (Fig. 2).

### Transcriptional regulation of endothelial cell morphology

During vascularization, endothelial cells acquire specific morphological features to form vascular structures.

Although vasculature morphology has been studied widely both in vivo and in vitro, no key transcriptional signal initiating these morphological changes has yet been identified. Endothelial specification and vascular morphological change are closely related processes that occur in a partially simultaneous or sequential manner. Thus, it is unclear whether common transcriptional factors are involved in these processes or whether vascular morphology is regulated by specific factors. We discuss several transcriptional factors, including Mef2, Ets, and Forkhead, that may play important roles in early vascular development [4, 21, 22].

#### Mef2 transcription factors

Myocyte enhancer factor 2 (Mef2) is a member of MADS box transcription enhancer factor family. Mef2 is an important cellular development regulator in multiple cell types in muscle, vascular, neural, and immune tissues [23–25]. In vertebrates, there are four MEF2 genes: Mef2a, Mef2b, Mef2c, and Mef2d. The expression of Mef2a, Mef2c, and Mef2d can be detected in the cardiovasculature network during early embryonic development [26, 27] and endothelial cells in vivo and in vitro [28, 29], pointing to a potential role for Mef2 in vascular development. Mef2a-null mice exhibit mitochondrial deficiency in cardiac muscle and perinatal lethality [30]. Mef2c-null mice show severe vascular malformations and die by E9.5 [28]. Loss of Mef2c does not affect the endothelial cell specification but inhibits smooth muscle cell differentiation, which causes the defects of vascular network. However, deletion of endothelial-specific Mef2c in mice does not result in obvious vascular defects in development [31]. Mice lacking Mef2b or Mef2d are viable and show no apparent abnormality [32, 33]. These phenotypes for each Mef2 gene deletion mutant appear to demonstrate distinct and partially overlapping functions among Mef2 members.

Mef2 factors have been demonstrated to regulate sprouting angiogenesis. In the presence of VEGF, these factors regulate the transcriptional activation of a Notch ligand Delta-like ligand 4 (Dll4) in endothelial cells [29]. Induced endothelial deletion of both Mef2a and Mef2c suppresses sprouting angiogenesis in mouse retina. In contrast, Mef2c has been shown to negatively regulate angiogenesis [34]. Mef2c overexpression inhibits VEGF-induced tube formation in HUVEC on collagen gel cultures, while Mef2 inhibition in a dominant-negative mutant enhances basal tube formation.

#### Ets and Foxc transcription factors

De Val et al. described a conserved endothelial cell-specific enhancer identified from the Mef2c gene [35, 36]. The enhancer contains a composite DNA binding site, referred to as the FOX:ETS motif. Etv2, also known as ER71 or Etsrp71, which is an ETS family transcription factor. Forkhead box C2 (Foxc2), also known as Mfh-1, is a member of the forkhead box (Fox) family of transcription factors. Etv2 in combination with Foxc2 binds to the FOX:ETS motif, leading to the activation of genes for endothelial specification and establishment. Endothelial gene enhancers or promoters, such as Mef2c, Flk1, Tie2, Tal1, Notch4, and VE-cadherin, contain FOX:ETS motifs and thus appear to be under the control of the combination of Etv2 and Foxc2.

Loss of Etv2 causes a complete lack of endothelial and blood cells whereby mutant mice die by E10.5 [2, 37], suggesting that Etv2 is an indispensable factor in vasculogenesis and hematopoiesis. Foxc2 knockout mice die prenatally and perinatally, with cardiovascular system, skeletal structure, and lymphatic vascular system malformations; however, major vessels remain [38, 39]. Foxc1 expression has been reported to overlap with that of Foxc2. Foxc1 knockout mice also die prenatally and perinatally, with similar abnormalities as mutants lacking Foxc2 [40–42]. Foxc1 and Foxc2 compound null mice die by E9.5 and display more severe cardiovascular system abnormalities compared with mice lacking either Foxc1 or Foxc2 [42–44]. Moreover, Foxc1 is also able to bind to the FOX:ETS motif [36]. These findings indicate redundant roles of Foxc1 and Foxc2 during vasculature development [45].

We previously demonstrated that the Mef2c enhancer with the FOX:ETS motif is activated in endothelial cell precursors derived from murine embryonic stem (ES) cells [46, 47]. Activation is induced continuously in differentiated endothelial cells with a flat polygonal shape, and cells with an elongated shape stimulated by a high concentration of VEGF. Therefore, activation of the FOX:ETS motif may participate in endothelial cell lineage specification, but it is not necessarily connected to changes in endothelial cell morphology.

#### Foxo transcription factors

The forkhead box O (Foxo) transcription factor family contains four members: Foxo1, Foxo3, Foxo4, and Foxo6. This family is generally associated with promoting cell cycle arrest as well as inducing apoptosis and oxidative stress resistance [48, 49]. High Foxo1 expression can be observed in developing embryonic vasculature. Foxo1-deficient mice can provide insight into the role Foxo1 plays in vascular morphology during embryonic development [50, 51]. While Foxo1-null embryos have differentiated endothelial cells, they exhibit severe vascular defects including underdevelopment of branchial arches and malformation of dorsal aorta, thus resulting in death near embryonic days 10.5–11. This phenotype is mimicked by endothelial cell-specific deletion of Foxo1 in mice [52], suggesting that Foxo1 expression in endothelial cells is required for vascular structure formation in vivo.

ES cell-derived Foxo1-deficient endothelial cells do not exhibit cell elongation in response to VEGF [50]. They show actin-microtubule cytoskeleton disorganization and fail to interact with smooth muscle cells [53], suggesting a potential role of Foxo1 in cytoskeletal remodeling and smooth muscle cell recruitment. Conversely, Foxo1 may be required for endothelial growth control during postnatal vascular development or in mature endothelial cells. Inducible endothelial cell-specific disruption of Foxo1 enhances endothelial proliferation and leads to hyperplastic vasculature during retinal angiogenesis in mice [54]. Foxo1 appears to confer quiescence in endothelial cells by reducing metabolic activity via suppressed Myc signaling. Combined deletion of Foxo1, Foxo3, and Foxo4 in mice causes hemangiomas, which is increasing along with aging process [55]. Foxo1 overexpression suppresses migration and matrigel tube formation in human umbilical vein endothelial cells (HUVEC) [56]. Moreover, Foxo1 may bind less strongly to the FOX:ETS motif than FoxC1 or FoxC2 [36]. These findings suggest that Foxo1 may regulate multiple discrete endothelial cell functions during vascularization. Although multiple factors have been demonstrated that appear to be regulated by Foxo1, including Ang2, CXCR4, PDGF-B, and Flk1 [56], the transcriptional targets involved with changes in endothelial morphology remain unclear.

Foxo1, Foxo3, and Foxo4 have highly conserved amino acids [57]. However, Foxo3- and Foxo4-null mice are viable and outwardly normal, with no detectable vascular formation abnormalities comparable to those observed in Foxo1-null mice [50, 51]. Foxo6-null mice also develop normally and do not show any vascular malformation [58]. The redundant functions of Foxo subtypes have been demonstrated with in vitro experiments. Foxo3 induction in Foxo1-deficient endothelial cells derived from ES cells alleviates morphological abnormalities during the late, but not early, differentiation stage [59]. Foxo3 as well as Foxo1 overexpression inhibits migration and tube formation in HUVEC [56]. Foxo3 may partially overlap with Foxo1 in regulating endothelial cell functions.

### Signaling regulation of endothelial cell morphology

VEGF is a well-known angiogenic factor that regulates various endothelial functions including survival, proliferation, migration, differentiation, and vascular permeability [60, 61]. The VEGF pathway interacts and associates with multiple pathways, such as PI3K-Akt, mTOR, and Notch signaling [62, 63], and transmits signals to cells during angiogenesis.

#### VEGF signaling

The signaling of VEGF and VEGFR2 plays an indispensable role in vascular development as well as regulates multiple angiogenic processes, including vascular growth and homeostasis [60, 64]. Heterozygous disruption of VEGF in mice causes abnormal blood vessel formation, resulting in embryonic death by E12 [65, 66]. VEGFR2-deficient mice die by E9.5 due to underdeveloped hematopoietic and endothelial cells [67]. These experiments identify the critical function of VEGF-VEGFR2 in vascular development. VEGF binds to and activates VEGFR2, leading to activation of intracellular signals such as protein kinase C (PKC), mitogen-activated protein kinase (MAPK), phosphatidyl inositol 3-kinase (PI3K)/Akt, also known as protein kinase B (PKB), and focal adhesion kinase (FAK). These signals regulate endothelial cell proliferation, migration, survival, and permeability [60]. VEGF has been reported to induce Mef2c expression and regulate endothelial cell functions such as migration or tube formation [68, 69].

Many studies demonstrate the important role VEGF plays in both physiological angiogenesis and pathological or therapeutic angiogenesis. Aberrant VEGF production in a tumor environment is induced through activation of hypoxia-inducible factor 1α (HIF-1α) mostly under hypoxia and results in the formation of disorganized and leaky tumor vessels [60, 70]. These abnormal vascular networks prevent the delivery of anti-cancer drugs to the tumor. Therefore, normalization of tumor vasculature has been proposed as a potential therapeutic strategy in cancer treatment [71–73]. VEGF has also been applied in tissue engineering to gain neovascularization due to its strong induction of angiogenesis [74, 75]. However, over-secretion of VEGF in myoblasts reportedly leads to hemangioma when transplanted into mouse muscle [76]. Although VEGF is required for vascular formation and maintenance, an appropriate level of VEGF in each environment is crucial.

**Fig. 3:**
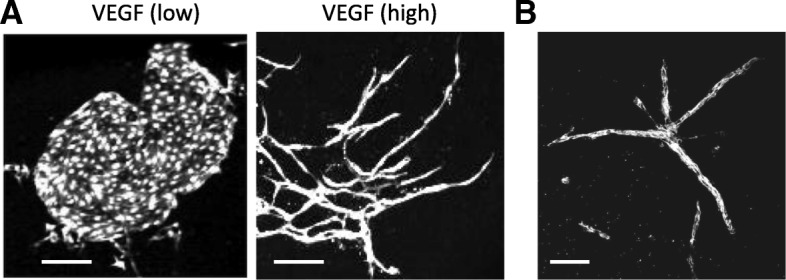
Elongation of endothelial cells derived from ES cells. **a** In the co-culture system with OP9 stromal feeder cells, ES-derived endothelial cells form a round colony in the presence of a low level of VEGF, which produced by OP9 cells (left panel). These cells form long elongated structures, when stimulated by a high level of VEGF (right panel). Scale bar 200 μm. **b** ES-derived endothelial cells form vessel-like structures in the 3D collagen gel culture. Scale bar 100 μm

We recently demonstrated that pharmacological inhibition of PI3K-Akt and mammalian target of rapamycin complex 1 (mTORC1) signaling can induce endothelial cell elongation without excess VEGF stimulation [47]. ES-derived endothelial cells require a low level of VEGF for growth. When stimulated by high levels of VEGF, these cells show over-growth and a shift from a flat, polygonal shape to a long, elongated shape (Fig. 3) [46, 47, 59]. However, PI3K-Akt or mTORC1 signaling inhibition also induces endothelial cell elongation in the presence of low levels of VEGF. These results indicate that PI3K-Akt and mTORC1 negatively regulate endothelial cell elongation.

#### PI3K-Akt signaling

PI3K-Akt signaling is activated by growth factors and angiogenic factors such as insulin, VEGF, and angiopoietin [77–79]. The serine/threonine protein kinase Akt is phosphorylated and activated by phosphoinositide-dependent kinase 1 (PDK1) when it binds to phosphatidylinositol-3,4,5-trisphosphate (PIP3) produced by PI3K. Phosphatase and tensin homolog deleted on chromosome 10 (PTEN) suppresses PI3K signaling through dephosphorylation of PIP3. Activated Akt negatively or positively regulates downstream targets to control various cellular functions including cell survival, proliferation, and metabolism. PI3K-Akt signaling has been shown to increase VEGF expression by producing the HIF-1α protein, thereby inducing angiogenesis [80, 81]. The binding of VEGF and VEGFR2 appears to increase mTORC1 activation through PI3K-Akt signaling, leading to endothelial cell survival and growth [77, 82, 83].

PI3K isoforms are divided into three classes: class I, class II, and class III [78, 84]. Blocking class IA PI3K signaling through general or endothelial cell-specific inactivation of p110α subunits in mice results in embryonic lethality by E12.5 due to severe vascular defects including an underdeveloped vascular plexus and poorly remodeled, enlarged vessels [85]. Endothelial cell-specific loss of class IA PI3K signaling through ablation of p85α and p85β subunits also causes embryonic lethality at E11.5 due to hemorrhaging [86]. These mice show normal phenotypes during early vasculogenesis but later show severe microvessel dilation and red blood cell congestion. General or endothelial cell-specific class II PI3K-C2α-deleted mice die between E10.5 and E12.5 due to vascular abnormalities, including microvessel dilation, hemorrhaging, and reduced branching [87]. These findings suggest that these signaling pathways are not involved in the initial stages of vascular development but are required for subsequent vascular remodeling and integrity.

Akt has three isoforms (Akt1, Akt2, and Akt3) that have partially overlapping and specific functions. Akt1 has been demonstrated to play a central role in angiogenesis [77] Akt1-deficient mice are viable but show reduced body weight [88, 89]. In vascular structure, loss of Akt1 causes decreased vascularization and reduced phosphorylation of endothelial nitric-oxide synthase (eNOS) in the placenta [89]. Postnatal deletion of endothelial-specific Akt1 in mice leads to delayed angiogenesis in mouse retina [90]. Endothelial coverage and radial outgrowth are also reduced in mouse retina. These findings indicate that PI3K and Akt are essential signaling molecules in vascular development.

Foxo1 is reportedly phosphorylated by PI3K-Akt signaling then translocalized from the nucleus to the cytoplasm, which results in suppression of its transcriptional activity [91, 92]. Inhibition of PI3K-Akt signaling by LY294002 or Akt inhibitor VIII induces endothelial cell elongation in the presence of low levels of VEGF [47]. In contrast, Foxo1-deficient endothelial cells fail to respond to PI3K-Akt inhibition even in the presence of excess VEGF. These findings imply that PI3K-Akt inhibition induces endothelial cell elongation by activating Foxo1. Thus, PI3K-Akt signaling appears to negatively regulate the elongation of endothelial cells. Ola et al. reported that PI3K signaling inhibition improves vascular defects in a mouse vascular malformation caused by blocking of bone morphogenetic protein (BMP) 9/10 or Activin receptor-like kinase 1 (Alk1) [92]. Loss of BMP9/10 or Alk1 increases Akt and Foxo1 phosphorylation in endothelial cells. These findings suggest PI3K-Akt signaling plays a key role in regulating endothelial cell morphology.

#### mTOR signaling

mTOR signaling is a crucial mediator in cell survival, proliferation, metabolism, and tumorigenesis [93]. mTOR is a serine/threonine protein kinase that forms two functionally distinct complexes, mTORC1 and mTORC2. The role of mTORC1 signaling in tumor angiogenesis is well understood. The GTP-bound active form of Ras homolog enriched in brain (Rheb) interacts and activates mTORC1, which drives VEGF secretion in tumor cells by inducing HIF-1α, promoting fragile tumor vessels [62, 77, 94]. Rheb is inactivated by tuberous sclerosis 1 (TSC1) and TSC2, which enhance conversion to the GDP-bound inactive form of Rheb due to its GAP activity. TSC2 is phosphorylated and inactivated by Akt [95, 96]. Thus, PI3K-Akt signaling is involved in mTORC1 activation.

Phenotypic analysis highlights critical players of mTOR signaling in embryonic development using general or endothelial cell-specific knockout mice. Regulatory-associated proteins of MTOR, complex 1 (Raptor) is an essential component of mTORC1. RPTOR independent companion of MTOR, complex 2 (Rictor) is an essential component of mTORC2. Raptor-deficient mice die early in development [97]; embryos appear to show proliferation defects during the blastocyst stage. Loss of endothelial cell-specific Raptor also results in embryonic death [98]. Conversely, mice lacking Rictor display a normal phenotype until E9.5, after which they die mid-gestation, around E11.5 [97, 99]. Endothelial cell specific-loss of Rictor also leads to embryonic death around E11.5–12.5 [98, 100]. The loss of Rictor has been shown to cause reduced or delayed peripheral vascularization in mice [100]. These findings suggest that mTORC1 and mTORC2 signaling is required for fetal development and embryonic angiogenesis.

We have previously demonstrated that mTORC1 inhibition by rapamycin or everolimus induces ES cell-derived endothelial cell elongation in the presence of low levels of VEGF [47]. This elongation requires mTORC2 and depends upon Foxo1. mTORC1 has been reported to inhibit mTORC2 signaling by activating p70 ribosomal protein S6 kinase 1 (S6K1), which phosphorylates Rictor [101, 102]. Therefore, the inhibition of mTORC1 may lead to endothelial cell elongation by compensating for mTORC2 signal activation. It is well known that mTORC2 signaling results in phosphorylation and deactivation of Foxo by activating Akt during regulation of cell proliferation and survival [97, 103–106]. However, Foxo1 is required to induce endothelial cell elongation during low VEGF conditions [47]. Although Foxo1 is a prevalent factor in endothelial morphology, mTORC1 inhibition in combination with high VEGF levels can induce endothelial cell elongation in a Foxo1-independent manner. Disruption of mTORC2 signaling by the genetic loss or decline of Rictor can inhibit the vascular assembly or angiogenic sprouting stimulated by VEGF in endothelial cells on matrix cultures [98, 107]. Thus, mTORC2 signaling in association with high VEGF levels appears to drive endothelial cell elongation independently of Foxo1.

mTORC2 can control actin cytoskeleton organization, which is linked to cell morphology. Knockdown of Rictor prevents actin fiber assembly and fibroblast cell spreading [108]. mTORC2 regulates the actin cytoskeleton through Rho GTPase Rac. On the other hand, downregulation of Rictor reduces phosphorylation of protein Kinase Cα (PKCα) and causes prominent organization of cytoplasmic actin fiber and reduced cortical actin in Hela cells [109]. Although the function of mTORC2 in actin organization may depend on cell type, these findings suggest that mTORC2 is a major factor in regulating the actin cytoskeleton. Downregulation of Rictor inhibits the actin stress fiber formation stimulated by VEGF in endothelial cells [107]. Endothelial cell elongation induced by PI3K-Akt or mTORC1 inhibition requires actin remodeling by activating Rho-ROCK signaling [46, 47]. Moreover, our recent study shows that dual inhibition of mTORC1/mTORC2 by KU0063794, but not mTORC1-specific inhibition by everolimus, remarkably impairs both actin and microtubule organization, inhibiting endothelial cell elongation [110]. The defects appear to result from disorderly microtubule distribution or stability. These findings suggest the mTOR signaling pathway is an important signaling node that modulates endothelial cell elongation by shaping the actin and microtubule cytoskeleton. Further studies are necessary to elucidate the mechanisms of mTOR signaling in endothelial cell morphological change.

#### Notch signaling

Four Notch receptors (Notch1, Notch2, Notch3, and Notch4), as well as five Notch transmembrane ligands of the Delta-Serrate-Lag (DSL) type, Jagged1 and 2 (Jag1 and Jag2), and Delta-like 1, 3, and 4 (Dll1, Dll3, and Dll4), are found in mammals [63, 111]. Notch signaling is involved in multiple stages of vascular development, including proliferation, migration, and arterial-venous endothelial cell fate determination [112]. Notch signaling is initiated by interactions between the Notch receptor and its ligand, which leads to the cleavage and release of the Notch intracellular domain (NICD). The NICD translocates to the nucleus and binds to a recombination signal binding protein for immunoglobulin kappa J region (RBP-j), also known as CBF1/Igkjrb/PBPjk. They then upregulate the expression of their target genes, hairy and enhancer of split (Hes) or Hes-related with YRPW motif (Hey). Hes1 suppresses PTEN expression, resulting in PI3K-Akt signal activation [113]. Further, non-canonical Notch signaling has been reported to interact with mTORC2 [114, 115]. NICD regulates cell survival through the activation of Akt, depending on mTORC2.

Notch signaling, together with VEGF, controls sprouting angiogenesis through endothelial cell specification. Endothelial cells are specialized to tip cells and stalk cells in sprouting angiogenesis [5, 116, 117]. Tip cells lead and guide blood vessel sprouts, and stalk cells follow tip cells and form sprout elongation. In tip cells, VEGF signaling induces the expression of Dll4, which binds to and activates Notch signaling in neighboring stalk cells and suppresses stalk cell VEGFR expression. As tip cells expressing Dll4 receive stronger VEGF stimulation, they acquire the higher motility and sprouting activity, resulting in further angiogenesis. This Dll4-Notch signaling is antagonized in stalk cells by Jagged1, which modulates the base of emerging vessel sprouts. Notch signaling attenuation or heterozygous Dll4 deletion in mice increases the number of tip cells and enhances cell proliferation, causing excessive vessel sprouting and branching defect [118, 119]. Thus, Notch signaling is essential in controlling angiogenic sprouting.

Loss of Notch1 in mice leads to embryonic lethality by E11.5 [120, 121], whereas Notch4-deficient mice are viable and exhibit no phenotypic defects [122]. However, deleting both Notch1 and Notch4 genes in mice causes embryonic lethality due to more severe vascular defects than Notch1 knockout mice [122]. Double-deletion mutants have normal vasculogenesis, but fail to perform vascular remodeling. This suggests a partially redundant function of Notch1 and Notch4 during vascular development. Similar findings were reported in RBP-j-deleted mice [122, 123]. Heterozygous deletion of Dll4 in mice also causes similar vascular defects to those in Notch1 and Notch4 double-deletion mutants, although vascular remodeling defects are less severe [123]. Hey1 and Hey2 as well as Hes1 and Hes5 show subtype redundancy in vascular development. Hey1-deficient mice develop normally [124], and Hey2-deficient mice do not show apparent embryonic vessel development defects but have postnatal cardiac hypertrophy [125]. Loss of both Hey1 and Hey2 leads to embryonic death by E11.5 due to defects of vascular development [124, 126]. In these mutants, early vasculogenesis is normal but large vessels do not form in the yolk sac and poor development of large vessels frequently occurs in the embryo, highlighting defects in vascular remodeling. Mice lacking either Hes1 or Hes5 exhibit no obvious abnormalities during vascular development, whereas general or endothelial-specific Hes1 deletion mutants on a Hes5-null background show defects in brain vascular remodeling [127].

In human arterial endothelial cells, VEGF induces Notch1 and Dll4 expression through the PI3K-Akt pathway [128]. Activation of Notch signaling using NICD or Hes1 expression enhances network and cord formation in a three-dimensional model, whereas blocking Notch signaling using a dominant-negative form of RBP-j partially inhibits the network formation stimulated by VEGF. Moreover, Notch1 signaling is activated by fluid shear stress in human aortic endothelial cells [129]. Fluid shear stress is a biophysical trigger of morphological change in endothelial cells, although flow-induced shape is not identical to the vessel-like elongation of ES cell-derived endothelial cells in response to VEGF (Fig. 3). It has been established that fluid flow induces each endothelial cells to elongate in parallel to the direction of flow and causes actin filament alignment [129, 130]. Reduced expression of Notch1 in vivo and in vitro has been shown to prevent endothelial cell elongation in response to flow as well as promote endothelial cell proliferation [129]. These findings suggest that Notch signaling plays an important role in modifying endothelial cell morphology.

### Vascular regeneration

Transcription factors and signaling molecules required for vascular regeneration in adulthood have been reported. The expression of Etv2 is very low or absent in adult, however, its expression is upregulated in endothelial cells following ischemic injury [131]. Deletion of endothelial-specific Etv2 impairs neovascularization in mouse hindlimb ischemia model. The overexpression of Etv2 improves vessel formation after ischemia. Moreover, ischemic injury upregulates Dll4 expression in microvascular endothelial cells of normoperfused muscles [132]. Dll4 inhibition in a soluble mutant impairs blood flow recovery and neovascularization after ischemia in muscle. On the other hand, Foxo transcription factor is reported to negatively regulate postnatal neovascularization. Deleting Foxo3 gene in mice causes the enhanced reperfusion and the increased capillary density in hind limb ischemia [56]. These factors are critical for vascular formation during embryonic development, moreover, involved in positively or negatively regulate vascular regeneration following injury.

## Conclusion

Comprehending the mechanisms regulating vascular structure formation is crucial to gain insight into both the physiological angiogenic process as well as diseases surrounding pathological angiogenesis. Abnormal or excessive angiogenesis is linked to increased tumor development [60, 70]. In diabetic patients, uncontrolled formation or deficiency of vessels, known as disordered angiogenesis, contributes to mortality and disability [133]. Furthermore, establishing functional vascular networks is key for tissue and organ regeneration in tissue engineering [74, 75].

Transgenic lines (Table 1) or cultured models (Fig. 3) help to visualize vascular structure or cell shape, facilitating evaluation of vascular morphology. However, the mechanisms modulating endothelial cell morphological change are not well understood compared with endothelial cell differentiation or proliferation. This may be due to the intricate behaviors of endothelial cells and the diverse roles played by angiogenic factors. Accurately classifying endothelial cell events during vascular development is difficult, as events occur in spatially and temporally similar or related contexts. Furthermore, as described in the literature, relevant factors and signaling molecules frequently have overlapping functions or associated interactions. Consequently, it may be even more important to investigate and reveal specific molecules or mechanisms associated with endothelial cell morphological change. A deeper understanding of vascular development holds promise for developing new therapeutics regulating vascular function.

**Table 1:** Mouse phenotypes

Disrupted gene		Phenotype	References
Mef2 transcription factors			
Mef2a		Perinatal death (cardiac sudden death), mitochondrial defects	[30]
Mef2b		Normal cardiac development	[32]
Mef2c		Embryonic death by day 9.5, cardiovascular defects, defects of smooth muscle cell differentiation	[28]
Mef2c (endothelial-specific deletion)		Promotion of vascular growth in oxygen-induced retinopathy	[31]
Mef2d		Resistance to cardiac hypertrophy induced by pressure overload	[33]
Ets and Foxc transcription factors			
Etv2		Embryonic death by day 10.5, defects of blood and vessel development	[2, 37]
Etv2 (endothelial-specific deletion)		No obvious phenotype in steady state condition	[131]
Foxc1		Prenatal and perinatal death, cardiovascular abnormalities, skeletal defects	[40–42]
Foxc2		Prenatal and perinatal death, cardiovascular and lymphatic abnormalities, skeletal defects	[38, 39]
Foxc1 and Foxc2		Embryonic death by day 9.5, more severe defects of cardiovascular and lymphatic development than Foxc1 or Foxc2-null mice	[41, 43, 44]
Foxo transcription factors			
Foxo1		Embryonic death by day 10.5–11, vasculature defects	[50, 51]
Foxo1 (endothelial-specific deletion)		Embryonic death by day 11, vasculature defects	[52]
Foxo3		Age-dependent infertility, abnormality of ovarian follicular development	[50, 51]
Foxo4		Normal	[50, 51]
Foxo6		Defects of memory consolidation	[58]
VEGF signaling			
VEGF (heterozygous deletion)		Embryonic death by day 12, abnormality of vascular development	[65, 66]
VEGFR2	VEGF receptor	Embryonic death by day 9.5, defects of hematopoietic and endothelial cell development	[67]
PI3K-Akt signaling			
p110α (general or endothelial-specific inactivation)	Class IA PI3K subunit	Embryonic death by day 12.5, vascular defects	[85]
p85α and p85β	Class IA PI3K subunit	Embryonic death by day 11.5, vascular defects, hemorrhage	[86]
PI3K-C2α (general or endothelial-specific deletion)	Class II PI3K subunit	Embryonic death by days 11.5–12.5, vascular defects, hemorrhage	[87]
Akt1		Growth retardation, reduction of vascularization in placenta	[88, 89]
Akt1 (endothelial-specific postnatal deletion)		Reduction of vascular development in retina	[90]
mTOR signaling			
Raptor	mTORC1 subunit	Embryonic death at early stages of development	[97]
Raptor (endothelial cell-specific deletion)		Embryonic death	[98]
Rictor	mTORC2 subunit	Embryonic death by day 11.5, growth arrest, placental abnormalities	[97, 99]
Rictor (endothelial cell-specific deletion)		Embryonic death by days 11.5–12.5, growth retardation, reduction of peripheral vascularization	[98, 100]
Notch signaling			
Notch1	Notch receptor	Embryonic death by day 11.5, delayed and disorganized somitogenesis	[120, 121]
Notch4	Notch receptor	Normal	[122]
Notch1 and Notch4		More severe phenotype than Notch1-null mice, defects of vascular remodeling	[122]
Dll4 (heterozygous deletion)	Notch ligand	Similar to phenotype of Notch1 and Notch4-null mice, defects of vascular remodeling	[123]
RBP-j	Notch transcriptional effector	Defects of vascular remodeling and somite formation	[123]
Hey1	Notch target gene	Normal	[124]
Hey2	Notch target gene	Cardiac hypertrophy after birth	[125]
Hey1 and Hey2		Embryonic death by days 9.5–11.5, defects of vascular remodeling, hemorrhage	[124, 126]
Hes1	Notch target gene	No obvious phenotype in vascular development	[127]
Hes5	Notch target gene	Normal	[127]
Hes1 and Hes5 (general or endothelial-specific deletion of Hes1 on Hes5-null background)		Defects of vascular remodeling in the brain	[127]

## Abbreviations

Alk1: Activin receptor-like kinase 1
BMP: Bone morphogenetic protein
COUP-TFII: Chicken ovalbumin upstream promoter-transcription factor II
Dll1, Dll3, and Dll4: Delta-like 1, 3, and 4
DSL: Delta-Serrate-Lag
eNOS: Endothelial nitric-oxide synthase
ES cells: Embryonic stem cells
FAK: Focal adhesion kinase
Fox: Forkhead box
Hes: Hairy and enhancer of split
Hey: Hairy/enhancer-of-split related with YRPW motif
HIF-1α: Hypoxia-inducible factor 1α
HUVEC: Human umbilical vein endothelial cells
Jag1 and Jag2: Jagged1 and 2
MADS: MCM1, Agamous, Deficiens, Serum-response factor
MAPK: Mitogen-activated protein kinase
Mef2: Myocyte enhancer factor 2
mTORC1: Mammalian target of rapamycin complex 1 or mechanistic target of rapamycin complex 1
NICD: Notch intracellular domain
PDK1: Phosphoinositide-dependent kinase 1
PI3K: Phosphatidyl inositol 3-kinase
PIP3: Phosphatidylinositol-3,4,5-trisphosphate
PKB: Protein kinase B
PKCα: Protein Kinase Cα
Prox1: Prospero homeobox 1
PTEN: Phosphatase and tensin homolog deleted on chromosome 10
Raptor: Regulatory associated protein of MTOR, complex 1
RBP-j: Recombination signal binding protein for immunoglobulin kappa J region
Rheb: Ras homolog enriched in brain
Rictor: RPTOR independent companion of MTOR, complex 2
S6K1: p70 ribosomal protein S6 kinase 1
TSC1: Tuberous sclerosis 1
VEGF: Vascular endothelial growth factor
